# Availability and readiness of health facilities providing services for other infectious diseases to treat neglected tropical diseases in Ethiopia: implications for service integration in high burden areas

**DOI:** 10.1186/s12913-024-11257-9

**Published:** 2024-07-26

**Authors:** Getahun Asmamaw, Tefera Minwagaw, Mastewal Samuel, Wondim Ayenew

**Affiliations:** 1 Unit of Social and Administrative Pharmacy, Department of Pharmacy, Arba Minch University, Arba Minch, Ethiopia; 2 Department of Pharmacy, Bahir Dar University, Bahir Dar, Ethiopia; 3 Department of Pharmacy, Wachemo University, Hassana, Ethiopia; 4 Department of Social and Administrative Pharmacy, School of Pharmacy, University of Gondar, Gondar, Ethiopia

**Keywords:** Availability, Service, Access to treatment, Infectious diseases, Neglected tropical diseases, Health facilities, Service integration, High-burden areas, Ethiopia

## Abstract

**Background:**

The burden of neglected tropical diseases (NTDs), HIV/AIDS, tuberculosis, and malaria pose significant public health challenges in Ethiopia. This study aimed to the explore service availability and readiness for NTD care among Ethiopian health facilities treating tuberculosis (TB), HIV/AIDS, and/or malaria.

**Methods:**

This study utilized secondary data from the Ethiopian Service Provision Assessment 2021–22 survey. The availability of services was calculated as the percentage of HIV/AIDS, tuberculosis, or malaria facilities providing NTD services. Facilities were considered highly prepared to manage any type of NTD if they scored at least half (> 50%) of the tracer items listed in each of the three domains (staff training and guidelines, equipment, and essential medicines). Descriptive statistics and logistic regression models were employed to present the study findings and analyze factors influencing facility readiness, respectively.

**Results:**

Out of 403 health facilities providing NTD care nationally, 179, 183, and 197 also offer TB, HIV/AIDS, and malaria services, respectively. The majority of TB (90.1%), HIV/AIDS (89.6%), and malaria (90.9%) facilities offer soil-transmitted helminth services, followed by trachoma (range 87–90%). The percentages of the aforementioned facilities with at least one trained staff member for any type of NTD were 87.2%, 88.4%, and 82.1%, respectively. The percentage of facilities with guidelines for any type of NTD was relatively low (range 3.7–4.1%). Mebendazole was the most widely available essential medicine, ranging from 69 to 70%. The overall readiness analysis indicated that none of the included facilities (TB = 11.9%; HIV/AIDS = 11.6%; and malaria = 10.6%) were ready to offer NTD care. Specifically, a higher level of readiness was observed only in the domain of medicines across these facilities. Hospitals had better readiness to offer NTD care than did health centers and clinics. Furthermore, a significant associations were observed between facility readiness and factors such as facility type, region, presence of routine management meetings, types of NTD services provided, and fixed costs for services.

**Conclusions:**

Ethiopian health facilities treating TB, HIV/AIDS, and malaria had an unsatisfactory overall service availability and a lack of readiness to provide NTD care. Given the existing epidemiological risks and high burden of TB, HIV/AIDS, malaria, and NTDs in Ethiopia, there is an urgent need to consider preparing and implementing a collaborative infectious disease care plan to integrate NTD services in these facilities.

## Background

Neglected tropical diseases (NTDs) represent a group of diverse infections that primarily affect populations living in poverty, particularly in tropical and subtropical regions. These diseases, including lymphatic filariasis, soil-transmitted helminthiasis, and schistosomiasis, often result in chronic disability, reduced productivity, and economic burdens on affected communities, [1]. Despite their significant impact on public health, NTDs can also increase the risk of coinfection and treatment failure with other infectious diseases, such as TB, HIV/AIDS, malaria, and even coronavirus, [2–4]. Examples are, individuals with lymphatic filariasis or onchocerciasis could have compromised immune systems, increasing their vulnerability to other infections, [5]. Schistosomiasis can cause liver damage and bladder cancer, complicating the treatment of other diseases, [6]. People with soil-transmitted helminthiasis may be malnourished or anemic, which can impair their reaction to vaccines or medicines, [7, 8].

Ethiopia, a country in the Horn of Africa, bears a considerable burden of NTDs, with various endemic diseases prevalent across different regions, [9–11]. Simultaneously, Ethiopia is also experiencing high rates of HIV/AIDS, TB, and malaria, posing a significant challenge to the country’s healthcare system, [12]. The World Health Organization (WHO) and through World Health Assembly (WHA) recommend an integrated provision of healthcare services for the control of the prevalence of infectious diseases particularly in high-burden areas, [13–15]. However, this approach has been applied to different extents in lower-income nations, with Ethiopia yet to begin, [16]. In terms of NTDs, Ethiopia has undertaken national efforts to strengthen vertical programs targeting specific types of NTDs such as onchocerciasis and trachoma, [17]. However, there is a lack of comprehensive service integration between NTDs with other correlated infectious diseases and even intraspecific NTDs, [11]. For example, despite the coexistence of NTDs with each of TB, HIV/AIDS and malaria diseases, there remains a significant gap in the integration of services for NTDs with any of such individual disease in the healthcare system, in Ethiopia, [16, 18]. The problem of inadequate integration of health services in Ethiopia stems from various factors. The country’s healthcare system faces resource constraints, including shortages of healthcare personnel, essential medicines, and diagnostic tools, which hinder the provision of comprehensive care for multiple diseases within a single facility, [11, 19]. Additionally, the fragmentation of healthcare delivery in Ethiopia arises from the separate vertical programs for HIV/AIDS, TB, malaria, and NTDs, resulting in fragmented service delivery and missed integration opportunities, [16, 18]. Furthermore, health system prioritization historically favors HIV/AIDS, TB, and malaria over NTDs, leading to the potential marginalization of NTD services within healthcare facilities primarily focused on addressing these priority diseases, [9]. A lack of coordination and collaboration among stakeholders responsible for managing HIV/AIDS, TB, malaria, and NTD programs further impedes efforts to integrate services effectively, [20, 21].

With respect to the distribution of NTD service provision in the Ethiopian healthcare system, NTD service is available at all levels of healthcare, [22]. Ethiopia has a three-tiered system of decentralized health service provision and control. Primary-level healthcare includes health posts, health centers, and primary hospitals. Health posts are designed to offer preventative care and health-promoting services. Health centers offer both preventative and curative services. Primary hospitals provide inpatient and outpatient services. Secondary and tertiary healthcare are composed of general hospitals and specialized hospitals, respectively. Private health facilities include clinics (all levels), diagnostic centers, pharmacies, and drugstores. Across the Ethiopian healthcare tier system, health institutions should provide infectious disease preventative treatments, conduct routine examinations and investigations, diagnose and treat HIV/AIDS, TB, malaria, and NTD patients, and refer severe cases to higher-level facilities, [22, 23].

Ensuring access to quality health services is one of the primary functions of a health system, [23, 24]. Thus, such a function is supposed to be satisfied by ensuring service availability, expanding physical facility reach, and improving preparedness capacity to provide services. According to the WHO, the service integration and collaborative program of HIV/AIDS, TB, malaria, and NTD services are strongly recommended. Hence, implementing collaboration strategies, such as joint coordination, bidirectional screening surveillance, and collaborative action for NTD and comorbid HIV/AIDS, TB, and malaria management, should be implemented, [25]. Moreover, the WHO-Service Availability and Readiness Assessment (SARA) sets basic requirements for health service delivery and readiness for particular health services, such as HIV/AIDS, TB, malaria, and NTDs, [24].

Currently, there is inadequate evidence of the capacity and readiness of Ethiopian healthcare facilities for managing infectious diseases, including HIV/AIDS, TB, malaria, and NTDs. According to the Ethiopian 2016 SARA report, Ethiopian health facilities demonstrated disparities in terms of service availability and preparedness for providing services for patients with NTDs, [23]. Specifically, the status, availability of services, and preparedness of the limited number of HIV/AIDS, TB, and malaria facilities to treat NTDs remain unrealized. Therefore, this study aimed to investigate the service availability and preparedness of HIV/AIDS, TB, and malaria facilities for managing NTDs in Ethiopia. Given the high burden of infectious diseases and the existence of challenges in the healthcare system such as resource and financial constraints, the findings of this study will contribute to a better understanding of the potential and extent of readiness of Ethiopian healthcare facilities for managing HIV/AIDS, TB, malaria, and NTD in an integrated manner. This could also benefit decision-makers and other relevant bodies to identify areas where action is needed.

## Methods

### Data source

A secondary data analysis of the Ethiopian Service Provision Assessment (ESPA) 2021-22 survey was used in this study, [22]. This survey was the second comprehensive survey in Ethiopia where the first survey was held in 2014. The Ethiopian Public Health Institute and the Ethiopian Ministry of Health conducted the recent ESPA survey. Financial and technical support was provided by the United States Agency for International Development and the International Classification Functioning Company (ICF International) under the DHS Program, respectively.

The ESPA survey was designed to collect essential data on the availability and readiness of basic healthcare services at each level of healthcare institutions in Ethiopia. Its purpose was to provide a comprehensive overview of the Ethiopian healthcare delivery system by assessing the availability and functions of basic components that are crucial for the provision of quality health services. Hence, the survey covered healthcare service areas such as child health, maternal and newborn care, family planning, sexually transmitted infections, HIV and AIDS, tuberculosis (TB), malaria, noncommunicable diseases, and NTDs.

### Sampling technique and sample size

Except for the Tigray region, the ESPA survey was a representative survey for all active formal health sectors in Ethiopia, [22]. A master list of health sectors obtained from the Ethiopian Ministry of Health included 25,711 functional health facilities in Ethiopia. The list consists of hospitals, health centers, health posts, and private clinics (specialty/higher clinics, medium clinics, and lower clinics). These facilities were managed by the government, private for-profit organizations, and nongovernmental organizations. The data collection tool used in the survey mainly consisted of three types of questionnaires such as facility inventory questionnaires, health provider interviews, and client exit interview questionnaires. Concerning the sampling design, the survey used stratified random sampling and systematic sampling approaches and 1,407 health facilities were selected. Thus, this study was designed to provide nationally representative results for each of Ethiopia’s eleven regional states, for overall facilities at the national level, and for facility types such as hospitals (including public and private), health centers, clinics, and health posts. The detailed method utilized in the ESPA survey can be found elsewhere, [22].

**Fig. 1:**
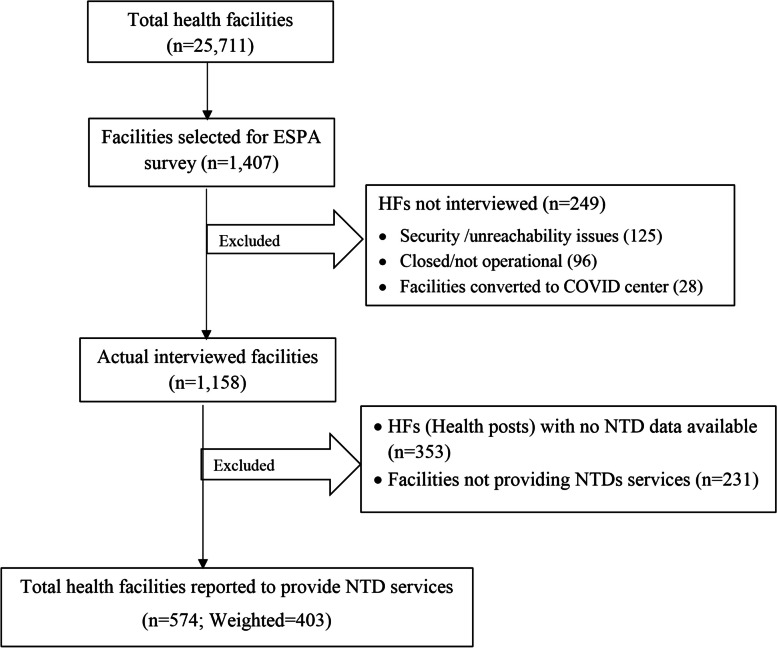
Selection of healthcare facilities (HFs) included in the recent study

This study used the facility inventory of the survey file to analyze data that make the unit of analysis at the facility level. The inclusion criteria for this study were that the health facility must be willing to participate in the survey, be open/operational on the day of the interview, have no security issues, must be reachable, not be converted to a coronavirus center, must provide NTD services, and must have collected data regarding the NTD on the recent dataset. As a result, out of the 1,407 facilities, facilities those that did not fulfill the inclusion criteria were excluded as follows, 125 facilities were excluded because of security /unreachability issues, 96 were closed/not operational on the day of data collection, 28 facilities were converted to coronavirus centers, 231 did not provide any type of NTD services, and all of the health posts (as the NTD data were unavailable in the dataset). Therefore, only 574 (weighted = 403) health facilities were included in the analysis in this study (Fig. 1).

### Measuring variables

#### Outcome variables

The first outcome of this study was “availability of NTD services indicators”. In this study, “NTD services” refer to providing both diagnosis and treatment for any type of NTD such as onchocerciasis, lymphatic filariasis, schistosomiasis, soil-transmitted helminths, trachoma, dracunculiasis, podoconiosis, and leishmaniasis. “Availability/availability of services” was defined as the percentage of TB, HIV/AIDS, or malaria facilities offering NTD services. TB/HIV/AIDS/malaria facilities are health facilities offering diagnosis and treatment services for either of TB, HIV/AIDS, or malaria diseases, respectively. This study adopted the WHO-SARA standards, and NTD services were considered “available” if the facility scored 100% of the availability of NTD service indicators in each of the three domains (each described below), [24]. The second outcome variable was “readiness”, and in this study, it was defined as the capacity of the health facility to offer NTDs. As recommended by the WHO-SARA, if a facility scored half or greater (≥ 50%) of the indicators in each of the three domains, it was classified as “high readiness.” Otherwise, if the facility scored less than half (< 50%) of the previously mentioned indicators in any of the three domains, it was deemed to have “low readiness”. Readiness was rated as an index that was classified into three domains as recommended by the WHO-SARA standards, [24]. The last outcome variable was “access to NTD care”. NTD services were considered accessible in the health facilities if they scored the highest for the both first (“available”) and second (“available”) variables.

In this study, the first domain was “staff and training” which has one indicator: the presence of at least one staff member who received a refresher training for the diagnosis and treatment of NTDs. Facilities with at least one staff member who received the abovementioned refresher training mentioned above within two years were classified as “Yes”; otherwise they were classified as “No”. The second domain included guidelines that had one indicator: the observed presence of national (and other) guidelines for NTD diagnosis and treatment. The facilities with such guidelines were classified as “Yes”; otherwise, they were classified as “No”. The third domain was key medicines that had one indicator: the observed availability of each first-line essential medicine such as sodium stibogluconate (SSG), ambisome, azithromycin, ivermectin, ivermectin + albendazole, praziquantel, and mebendazole at the facility. The variation in the disease burden of each NTD type in the country could determine the type of medicines facilities are holding. Therefore, in this study, the facilities reported the availability of at least one of seven NTD medicines were classified as “Yes”; otherwise, they were classified as “No”.

Based on the WHO-SARA standard, the responses were pooled into an index score to generate an aggregate score. The index score was computed by adding each available indicator and dividing it by the total number of indicators in that domain. To determine the readiness, the calculated index scores for each of the three domains were summed up once again and divided by the total number of domains (three). During analysis, equal weights were given for each domain and each indicator within the domains. Facilities that scored 50% or above were categorized as “high readiness” to provide NTD services.

1 $${\rm{Index}}\,{\rm{score}}\,\left( {{\rm{IS}}} \right){\rm{ = (}}\sum {{{\rm{Y}}_{\rm{i}}}} {\rm{)/}}{{\rm{n}}_{\rm{i}}}$$

2 $${\rm{High}}\,{\rm{readiness = (}}\sum {{\rm{I}}{{\rm{S}}_{\rm{i}}}} {\rm{)/}}{{\rm{N}}_{\rm{i}}} \times {\rm{ 100,}} \ge {\rm{50\% }}$$

*where, Y = number of present indicators; n = total number of indicators in the domain; i = types of domains; and N = total number of domains;*

The target of service availability was 100%, meaning that each domain accounted for one-third of (100/3 = 33.3%) of the index score. Therefore, the share percentage of each indicator in each domain was calculated by dividing 33.3% by the number of indicators for each domain. Hence, to calculate the domain-specific readiness, facilities scored at least half of 33.3% (≥ 16.7%) and were categorized as “high readiness” for specific domains. The cutoff values employed in this study were also utilized in other studies to dichotomize the outcome variables, [26–28].

#### Independent variables

The facility type was categorized as “hospital,” “health center,” or “clinic”; the place of residence was categorized as “urban” or “rural”; the managing authority was categorized as “public” or “private”; the region was categorized as one of ten regional states (“Afar”, “Amhara”, “Oromia”, “Somali”, “Benishangul Gumuz”, “South Nations Nationalities and People (SNNP)”, “Sidama”, “Gambela”, “Harari”) or two city administrations(“Addis Ababa” or “Dire Dawa”); routine management meetings were categorized as “performed” or “not performed”; external supervision was categorized as “received” or “not received”; external sources of revenue were categorized as “none,” “government” or “nongovernment”; user fees were categorized as “fixed for all services” for facilities with a fixed cost for all types of services the clients would receive otherwise were categorized as “separate”; for facilities reported to have any type of health insurance scheme were categorized as “accepted” or “not accepted”; and facilities reported to provide any type of NTD services were categorized as “available”; otherwise, they were categorized as “not available”.

### Data analysis

In this study, all estimates were sample-weighted to adjust for nonresponses and sampling disproportionality. The coded data were analyzed using STATA 14.2, statistical software. The study utilized both the descriptive and inferential statics approaches. All estimates were performed for each of the three health facilities (TB, HIV/AIDS, and malaria facilities) separately. In the descriptive analysis, the summary of the frequency and percentage of outcome variables was presented with tables and graphs as needed.

Logistic regression models were applied to estimate the effects of explanatory (health facility) variables (facility type, place of residence, region, managing authority, routine management meeting, external supervision, an external source of revenue, type of user-fees, presence of health insurance) on the likelihood of accessing to treatment and facility readiness for NTD services in each of the three facilities. Unfortunately, none of the facilities achieved our definition of “access to NTD care”, so we could not perform the inferential statistics for this outcome variable. Therefore, bivariate and multivariate logistic regression models were performed sequentially for the outcome variable “readiness”. First, a bivariate regression model was applied, and explanatory variables with *P* ≤ 0.25 were included in the multivariate logistic regression. Furthermore, similar steps of the aforementioned inferential statistical models were used separately, to estimate the effect of facility variables on each of the three predetermined specific domains of outcome variables (staff training, guidelines, and essential medicines). A significant association was detected if the *P*-value at the 95% confidence interval (CI) was < 0.05. The adjusted odds ratio (AOR) was also estimated for covariates fitted for multivariate logistic regression. Multicollinearity test was also performed for variables included in the model, that should not exceed 4.0. In this study’s analysis, all variables included in the model had variance inflation factors (VIFs) < 4.0, and the mean VIF for all variables was 1.86, suggesting that no harmful collinearity occurred in the model.

## Results

### Distribution characteristics of healthcare facilities

In the Ethiopian SPA 2021-22 survey, a total of 262, 259, and 340 health facilities reported providing interventions for TB, HIV/AIDS, and malaria-related services, respectively (Table 1). Nationally, 403 facilities were reported to offer services for any type of NTD. Overall, 179 (69.1%), 183 (69.8%), and 197 (57.9%) of all TB, HIV/AIDS, and malaria facilities offer diagnosis, respectively, and treatment for NTDs, respectively. Among the facilities that reported providing NTD care, the majority (TB facilities = 74.3%; HIV/AIDS facilities = 73.8%; malaria facilities = 64%) were health centers. The majority of TB facilities (53.1.7%) and HIV/AIDS facilities (51.9%) providing NTD services were located in rural settings, whereas more than half of the malaria facilities were located in urban settings. Concerning the managing authority, nearly three-fourths (> 70%) were publicly owned. Health facilities from the Oromia and Amhara regional states account for more than half of the included facilities. The percentages of TB, HIV/AIDS, and malaria facilities that had routine management meetings were 95%, 93.4%, and 87.8%, respectively. However, the percentage of facilities that had received external supervision was less than 35% in each of the three health facilities.

**Table 1:** Distribution characteristics of TB, HIV/AIDS, and malaria facilities reported to provide NTD services for Ethiopia SPA 2021-22 (*n* = 179,183 and 197, respectively)

	Distribution of NTD services in:		
	TB facilities (*N* = 179/259)	HIV/AIDS facilities (*N* = 183/262)	Malaria facilities (*N* = 197/340)
	*n* (%)		
**Facility type**			
Hospitals	20 (11.2)	20 (10.9)	20 (10.2)
Health centers	133 (74.3)	135 (73.8)	126 (64.0)
Clinics	26 (14.5)	28 (15.3)	51 (25.9)
**Managing authority**			
Public	147 (82.1)	149 (81.4)	140 (71.1)
Private	32 (17.9)	34 (18.6)	57 (28.9)
**Residence**			
Urban	84 (46.9)	88 (48.1)	105 (53.3)
Rural	95 (53.1)	95 (51.9)	92 (46.7)
**Region**			
Afar	1 (0.6)	2 (1.1)	2 (1.0)
Amhara	43 (24.0)	43 (23.5)	48 (24.4)
Oromia	65 (36.3)	67 (36.6)	69 (35.0)
Somali	8 (4.5)	8 (4.4)	8 (4.1)
Benishangul Gumuz	1 (0.6)	1 (0.5)	1 (0.5)
SNNP	34 (19.0)	35 (19.1)	37 (18.8)
Sidama	9 (5.0)	9 (4.9)	10 (5.1)
Gambela	2 (1.1)	2 (1.1)	4 (2.0)
Harari	1 (0.6)	1 (0.5)	1 (0.5)
Addis Ababa	13 (7.3)	13 (7.1)	15 (7.6)
Dire Dawa	2 (1.1)	2 (1.1)	2 (1.0)
**External supervision**			
Received	57 (31.8)	56 (30.6)	61 (31.0)
Not received	122 (68.2)	127 (69.4)	136 (69)
**Routine management meetings**			
Performed	170 (95.0)	171 (93.4)	173 (87.8)
Not performed	9 (5.0)	12 (6.6)	24 (12.2)
**User fees**			
Fixed for all services	44 (24.6)	37 (20.2)	44 (22.3)
Separate for each service	135 (75.4)	146 (79.8)	153 (77.7)
**Health insurance**			
Accepted	128 (71.5)	130 (71)	121 (61.4)
Not accepted	51 (28.5)	53 (29.0)	76 (38.6)
**External source of revenue**			
Government	120 (67.0)	121 (66.1)	117 (59.4)
Other than government	7 (3.9)	9 (4.9)	11 (5.6)
None	52 (29.1)	53 (29.0)	69 (35.0)
**Types of NTD service**			
Onchocerciasis	94 (52.5)	97 (53.0)	102 (51.8)
Lymphatic filariasis	96 (53.4)	98 (53.6)	101 (51.3)
Schistosomiasis	124 (69.3)	127 (69.4)	137 (69.5)
Soil-transmitted helminths	161 (90.1)	164 (89.6)	179 (90.9)
Trachoma	158 (88.1)	164 (89.6)	172 (87.3)
Dracunculiasis	70 (39.2)	71 (38.8)	72 (36.5)
Podoconiosis	75 (41.7)	78 (42.6)	81 (41.1)
Leishmaniasis	83 (46.2)	84 (45.9)	91 (46.2)
**National**^a^	179 (69.1)	183 (69.8)	197 (57.9)

^a^National percentage of selected facilities offering any type of NTDs

Regarding the availability distribution of specific NTD services, the majority of TB, HIV/AIDS, and malaria facilities offer services for the management of soil-transmitted helminths (ranges 89–91%) followed by trachoma (ranges 87–90%). The lowest proportion of services provided by the respective facilities was for dracunculiasis, which ranged from 36 to 39.5% for each of the three health facilities providing NTD services (Table 1).

### Availability of basic service domains for NTD care

As shown in Table 2, the percentages of TB, HIV/AIDS, and malaria facilities with at least one trained staff member for any type of NTD were 156 (87.2%), 162 (88.4%), and 161 (82.1%), respectively. In terms of specific NTDs, staff trained for the treatment of trachoma account for the majority, followed by soil-transmitted helminths in each facility. However, less than 10% of facilities had a trained staff for the management of leishmaniasis in the respective healthcare facilities. The percentage of facilities having guidelines for any type of NTD was relatively low (ranges 3.7–4.1%). According to the data on the availability of essential medicines for NTD treatment, mebendazole was the most readily available medicine, ranging 69–70% in TB, HIV/AIDS, and malaria facilities. However, SSG, AmBisome, ivermectin, and ivermectin + albendazole were only available in fewer than 20% of the respective facilities.

**Table 2:** Availability of basic domains that support the provision of NTDs among TB, HIV/AIDS, and malaria facilities reported to provide NTD services

	Availability of NTD services in:		
	TB facilities	HIV/AIDS facilities	Malaria facilities
	*n* (%)		
**Have staff trained in**			
Onchocerciasis	17 (9.5)	19 (10.4)	19 (9.6)
Lymphatic filariasis	17 (9.5)	17 (9.2)	17 (8.6)
Schistosomiasis	18 (10.1)	18 (9.8)	19 (9.9)
Soil-transmitted helminths	27 (15.1)	28 (15.1)	26 (13.2)
Trachoma	45 (25.1)	47 (25.7)	47 (23.9)
Dracunculiasis	8 (4.5)	8 (4.4)	8 (4.1)
Podoconiosis	14 (7.8)	14 (7.7)	14 (7.1)
Leishmaniasis	10 (5.6)	11 (6.1)	11 (5.7)
**Have guidelines on**			
Onchocerciasis	0 (0.2)	0 (0.2)	0 (0.2)
Lymphatic filariasis	1 (0.7)	1 (0.7)	1 (0.6)
Schistosomiasis	1 (0.7)	1 (0.7)	1 (0.7)
Soil-transmitted helminths	0 (0)	0 (0)	0 (0)
Trachoma	1 (0.6)	1 (0.5)	1 (0.5)
Dracunculiasis	2 (0.9)	2 (0.8)	2 (0.8)
Podoconiosis	2 (0.9)	2 (0.8)	2 (0.8)
Leishmaniasis	0 (0.1)	0 (0.1)	0 (0.1)
**Have essential medicines**			
Sodium stibogluconate (SSG)	20 (11.3)	20 (11.1)	22 (11.1)
AmBisome	4 (2.0)	4 (2.0)	5 (2.6)
Azithromycin	76 (42.2)	76 (41.8)	73 (37)
Ivermectin	14 (7.6)	14 (7.4)	15 (7.7)
Ivermectin + albendazole	6 (3.4)	6 (3.4)	6 (3)
Praziquantel	79 (43.9)	80 (43.7)	78 (39.6)
Mebendazole	124 (69.3)	127 (69.6)	125 (63.2)

### Readiness of health facilities to offer NTD care

**Fig. 2:**
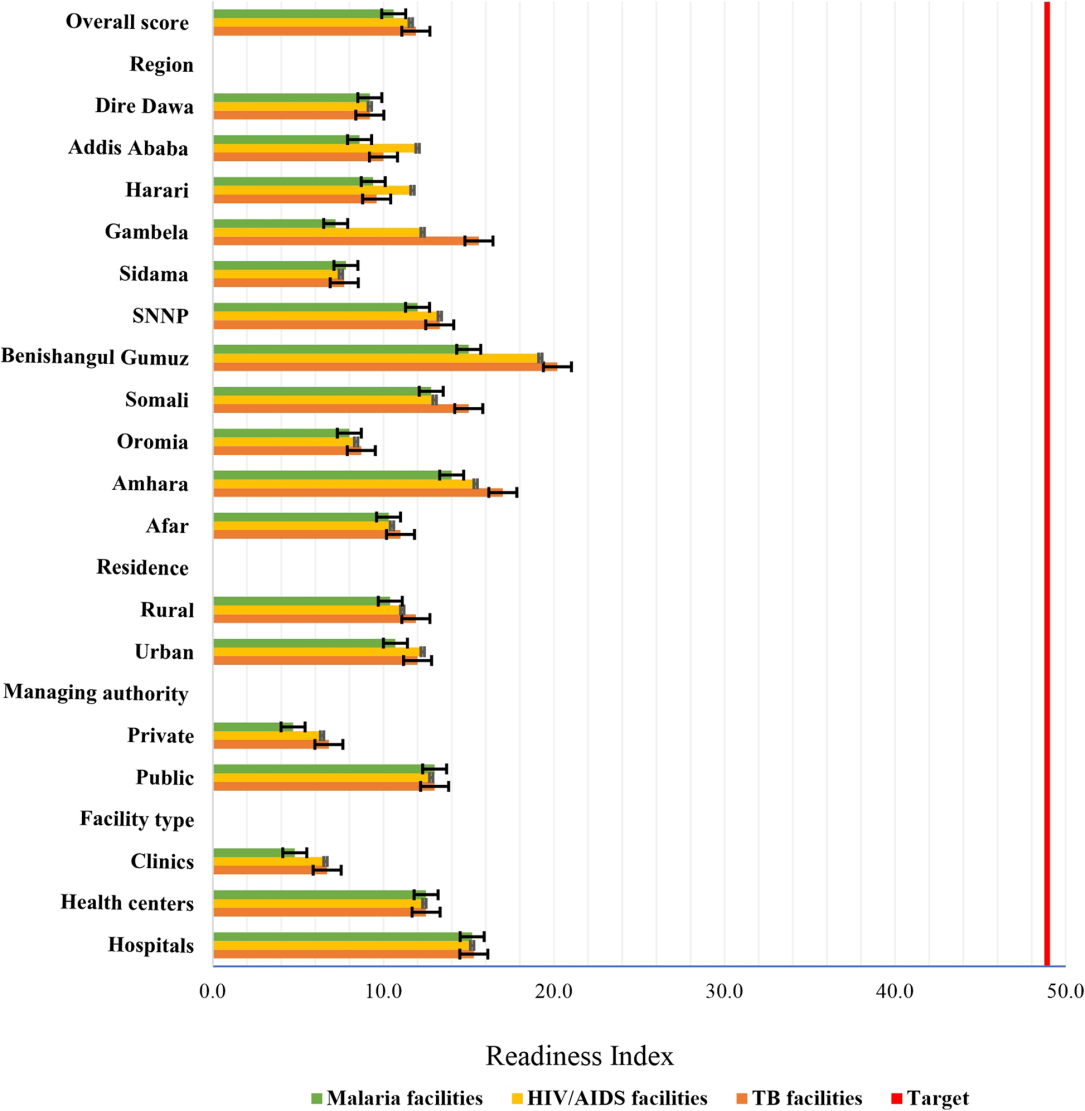
Percentage didtribution of facility readiness for treatment of NTDs among TB, HIV/AIDS, and malaria facilities reported to provide NTD services. (The vertical red line represents the cutoff value, beyond which facilities are considered to have low read

The overall readiness analysis indicated that none of the included facilities were ready (TB = 11.9%; HIV/AIDS = 11.6%; and malaria = 10.6%) to offer NTD treatment. This figure further highlights the total lack of access to NTD care among the studied health facilities (Fig. 2). Concerning the specific domains, none of the facilities were ready to offer NTD treatment in terms of trained staff except for health centers providing HIV/AIDS care, accounting for a readiness level of 17.2% (Table 3). In terms of the medicine domain, except for malaria facilities in Gambela, other facility characteristics were ready to offer NTD treatment accounting for the overall scores of 20.9%, 20.7%, and 22.1% in TB, HIV/AIDS, and malaria facilities, respectively.

**Table 3:** The domain-specific and total readiness of TB, HIV/AIDS, and malaria facilities reported to offer NTD services, Ethiopia SPA 2021-22

Variables	TB facilities (*N* = 179)				HIV/AIDS facilities (*N* = 184)				Malaria facilities (*N* = 197)			
	Staff & trainings	Guidelines	Medicines	Total mean score	Staff & trainings	Guidelines	Medicines	Total mean score	Staff & trainings	Guidelines	Medicines	Total mean score
	*n* [index %]			% (SD)	*n* [index %]			% (SD)	*n* [index %]			% (SD)
**Facility type**												
Hospitals	23 [8.6]	23 [0.5]	23 [36.6]	15.3 (9.0)	24 [8.4]	24 [0.5]	24 [36.7]	15.2 (8.9)	24 [8.4]	24 [0.5]	24 [36.3]	15.2 (8.9)
Health centers	175 [9.8]	175 [0.4]	175 [27.3]	12.5 (9.5)	180 [17.2]	180 [0.4]	180 [27]	12.4 (9.5)	170 [10.0]	170 [0.4]	170 [27]	12.5 (9.7)
Clinics	52 [0.7]	52 [0.1]	34 [19.8]	6.7 (10.1)	80 [1.3]	78 [0.03]	45 [19.3]	6.6 (9.3)	146 [0.8]	145 [0.03]	76 [14.1]	4.8 (8.8)
**Managing authority**												
Public	194 [9.7]	194 [0.4]	194 [28.6]	13.0 (9.6)	203 [9.3]	203 [0.4]	201 [28.6]	12.8 (9.5)	193 [9.6]	193 [0.4]	191 [28.6]	13.0 (9.7)
Private	56 [1.2]	57 [0.1]	38 [19.0]	6.8 (9.3)	80 [1.7]	80 [0.1]	47 [18]	6.4 (8.8)	147 [1.0]	147 [0.1]	79 [13.3]	4.7 (8.5)
**Residence**												
Urban	121 [5.5]	121 [0.2]	106 [29.7]	12.0 (10.7)	136 [5.4]	136 [0.2]	109 [30.5]	12.3 (10.5)	184 [4.2]	181 [0.1]	129 [30]	10.7 (10.8)
Rural	129 [10]	129 [0.5]	126 [24.8]	11.9 (9.0)	146 [8.8]	146 [0.5]	140 [23.5]	11.1 (9.0)	159 [8.0]	159 [0.4]	141 [21.7]	10.4 (9.3)
**Region**												
Afar	4 [2.5]	4 [0.0]	4 [30.2]	11.0 (6.5)	6 [1.7]	6 [0.0]	5 [29.4]	10.5 (6.3)	6 [1.6]	6 [0.0]	5 [28.7]	10.3 (6.3)
Amhara	60 [11.3]	60 [0.1]	53 [36.7]	17.0 (9.4)	67 [10.5]	67 [0.9]	59 [33.6]	15.4 (10.0)	81 [8.8]	81 [0.7]	65 [30.5]	14.0 (10.5)
Oromia	93 [2.8]	93 [0.0]	87 [23.0]	8.7 (6.5)	99 [3.0]	99 [0.1]	92 [22.5]	8.4 (6.4)	117 [2.4]	118 [0.1]	92 [21.3]	8.0 (7.5)
Somali	10 [10.9]	10 [0.1]	10 [34.0]	15.0 (12.3)	15 [8.4]	15 [0.1]	13 [29.6]	13.0 (12.0)	15 [8.4]	15 [0.1]	13 [29.4]	12.8 (12.0)
Benishangul Gumuz	2 [19.2]	2 [0.0]	2 [41.5]	20.2 (12.6)	2 [14.9]	2 [0.0]	2 [41.5]	19.2 (12.2)	7 [5.6]	7 [0.0]	3 [33.8]	15.0 (12.7)
SNNP	42 [16.5]	42 [0.1]	42 [23.1]	13.3 (11.9)	53 [13.1]	53 [0.1]	45 [24.1]	13.3 (11.5)	56 [12.3]	56 [0.1]	48 [21.7]	12 (11.7)
Sidama	12 [3.7]	12 [0.6]	10 [18.7]	7.7 (6.4)	12 [3.7]	12 [0.5]	3 [25.4]	7.5 (6.5)	13 [3.6]	13 [0.7]	11 [18.9]	7.8 (6.4)
Gambela	3 [16.9]	3 [1.3]	2 [30.1]	15.6 (12.5)	4 [12.7]	4 [1.0]	11 [18.9]	12.3 (11.6)	8 [6.0]	8 [0.4]	6 [16.0]	7.2 (9.7)
Harari	1 [3.0]	1 [0.0]	1 [25.2]	9.6 (10.9)	1 [3.7]	1 [0.0]	1 [30.6]	11.7 (10.9)	1 [2.5]	1 [0.0]	1 [24.6]	9.4 (10.7)
Addis Ababa	21 [3.0]	21 [0.7]	18 [26.0]	10.0 (11.3)	21 [3]	21 [0.7]	16 [30.7]	12 (11.3)	32 [1.9]	32 [0.5]	23 [22.5]	8.6 (10.8)
Dire Dawa	3 [0.6]	2 [0.0]	2 [26.9]	9.2 (5.8)	3 [0.0]	3 [0.0]	2 [27.5]	9.2 (5.8)	3 [0.5]	3 [0.0]	2 [26.9]	9.2 (5.7)
**Overall score (SD)**	7.8 (16.2)	0.4 (2.9)	27.1 (20.9)	11.9 (9.8)	7.2 (15.5)	0.3 (2.7)	26.6 (20.7)	11.6 (9.7)	5.9 (14.4)	0.3 (2.5)	24.2 (22.1)	10.6 (10.0)

Despite this low proportion of readiness, hospitals that provide either TB, HIV/AIDS or malaria services had better readiness to offer management for NTDs than did health centers and clinics (Table 3). Lower readiness was observed in facilities located in rural areas and privately owned facilities in the respective healthcare facilities. Furthermore, when compared to other regional states, the Benishangul Gumuz and Amhara regional states showed a higher level of readiness for NTD management.

### Factors associated with facility readiness to offer NTD care 

As shown in Table 4, because there was no overall readiness among healthcare facilities, an adjusted logistic regression model was performed between the independent variables and the likelihood of facility readiness in terms of specific readiness domains. Two variables namely external supervision, and type of NTD service (onchocerciasis, lymphatic filariasis, dracunculiasis, and podoconiosis) were excluded from the logistic regression models due to their harmful collinearity, in each of the three of facilities. Additionally, across the three facilities, four variables namely managing authority, health insurance, external source of revenue, and type of NTD service (soil-transmitted helminths, trachoma, and leishmaniasis), were excluded from multivariate logistic analysis because they had a P-value of > 0.25 in the bivariate regression. Moreover, some variables excluded from the multivariate analysis were facility- and domain specific. Thus, the predictor variables “region” and “routine management meetings” were excluded from the TB and malaria facilities [guideline domain], respectively. In HIV/AIDS facilities, variables such as facility type, residence, region, routine management meetings, and type of NTD service (schistosomiasis) were excluded when determining the factors associated with facility readiness in terms of guideline NTD services.

**Table 4:** A multivariate regression analysis of domain-specific readiness to offer NTD treatment among TB, HIV/AIDS, and malaria facilities, Ethiopia SPA 2021-22

Variables	TB facilities			HIV/AIDS facilities			Malaria facilities		
	Staff & trainings	Guidelines	Medicines	Staff & trainings	Guidelines	Medicines	Staff & trainings	Guidelines	Medicines
	AOR [95% CI]								
**Facility type [ref: clinics]**									
Hospitals	1.4 [0.4–4.5]	0.1 [0-2.3]	4.6 [1.5–14.0]*	1.3 [0.4–4.5]	**-**	1.0*	1.6 [0.5–4.9]	0 [0-1.4]	6.8 [2.6–17.7]**
Health centers	2.3 [0.7–7.8]	0.9 [0.1–11.7]	1.2 [0.4–3.7]	2.3 [0.6-8]	**-**	1.0	2.8 [0.9–8.9]	0.8 [0.1–10.9]	1.8 [0.7–4.7]
**Residence [ref: Urban]**									
Rural	1.6 [0.9–2.8]	1.1 [0.1–8.5]	1.3 [0.8–2.1]	1.6 [0.9–2.8]	-	0.6 [0.1–4.5]	1.6 [0.9–2.9]	1.6 [0.2–14.5]	1.3 [0.8–2.1]
**Region [ref: Addis Ababa]**									
Afar	0.7 [0.1–6.1]	**-**	2.5 [0.4–15.4]	0.6 [0.1–5.5]	**-**	1.9 [0.4–9.1]	0.6 [0.1–5.3]	1.1 [0.1–19]	1.5 [0.4–6.4]
Amhara	1.9 [0.7–4.8]	**-**	4.5 [1.7–12.2]*	1.9 [0.8–4.9]	**-**	4.2 [1.5–11.3]*	2 [0.8-5]	1.0	4.2 [1.6–11.1]*
Oromia	0.4 [0.1–1.3]	**-**	1.1 [0.5–2.4]	0.4 [0.1–1.3]	**-**	1 [0.4–2.2]	0.4 [0.1–1.4]	1.0	1 [0.5–2.3]
Somali	2.6 [0.8–8.1]	**-**	3.1 [1-10.1]	2.8 [0.9–8.4]*	**-**	3 [0.9–9.9]	3.1 [1-9.4]	1.0	3.7 [1.1–12.1]*
Benishangul Gumuz	4.2 [1.6–10.8]**	**-**	2.5 [0.2–29.4]	4.3 [1.7–11.2]**	**-**	2.4 [0.2–28]	1.0**	1.0	2.6 [0.2–30.5]
SNNP	4.2 [1.3–13.8]**	**-**	0.8 [0.3–1.9]	4.3 [1.7–11.2]**	**-**	0.8 [0.3–1.8]	4.3 [1.7–11]*	1.6 [0-68.5]	0.8 [0.4–1.9]
Sidama	1.5 [0.3–8.8]	**-**	1.7 [0.5–5.3]	4.4 [1.3–14.3]	**-**	1.5 [0.5–4.9]	4.2 [1.3–13.5]	1.0	1.2 [0.4–3.6]
Gambela	1.7 [0.3–6.8]*	**-**	0.3 [0.1–1.4]	1.9 [0.3–11.1]	**-**	0.3 [0.1–1.4]	1.7 [0.3–9.5]	1.0	0.3 [0.1–1.4]
Harari	1.0	**-**	1.8 [0.5–5.9]*	1.0	**-**	1.5 [0.5–5.1]	1.0	1.0	2 [0.6–6.7]*
Dire Dawa	1.0	**-**	0.5 [0.2–1.2]	0.8 [0.2–2.8]	**-**	0.4 [0.2–1.1]*	1 [0.3–3.5]	8.7 [0.2-430.5]	0.5 [0.2–1.2]*
**User fees [ref: Separate for each service]**									
Fixed for all services	1.4 [0.8–2.4]	14.1 [1.5–129]*	0.8 [0.4–1.3]	1.5 [0.9–2.6]	1.0**	0.8 [0.5–1.3]	1.5 [0.9–2.6]	55.3 [2-157.3]*	0.7 [0.4–1.3]
**Routine management meetings [ref: Performed]**									
Not performed	0.8 [0.2–3.2]	1.1 [0.5–2.4]	0.5 [0.2–1.7]	0.7 [0.2–2.8]	-	0.5 [0.2–1.6]	0.5 [0.1-2]	-	0.5 [0.2–1.3]
**Types of NTD service offered [ref: No]**									
Schistosomiasis	2.7 [1.3–5.7]*	1.4 [0.1–13]	0.9 [0.5–1.7]	3.1 [1.4–6.7]*	-	0.9 [0.5–1.6]	2.9 [1.4-6]*	3.2 [0.1–76.1]	0.9 [0.5–1.6]

- :*P*-value ≥ 0.25 hence, the variables were not included in the Multivariate analysis; *AOR *Adjusted Odds ratio, *Gov’t* Government

*:*P* < 0.05

**:*P* < 0.001

Even though variability was observed in three facilities, the factors significantly associated with the likelihood of facility readiness were facility type, residence, region, routine management meetings, and type of NTD service (schistosomiasis) among the three facilities. In TB facilities, the readiness in terms of trained staff was associated with region (Benishangul Gumuz [AOR = 4.2, 95% CI; 1.6–10.8]; SNNP [AOR = 4.2, 95% CI; 1.3–13.8]; Gambela [AOR = 1.7, 95% CI; 0.3–6.8])) and type of NTD service provided by the facility (schistosomiasis [AOR = 2.7, 95% CI; 1.3–5.7]). In terms of the guideline domain, the facility having a fixed cost for all types of services was significantly associated [AOR = 14.1, 95% CI; 1.5–129]). In regard to the medicine domain, facility type (hospitals [AOR = 4.6, 95% CI; 1.5–14.0]) and region (Harari [AOR = 1.8. 95% CI; 0.5–5.9]) were significantly associated.

Concerning HIV/AIDS facilities, the readiness in terms of trained staff was associated with the region (Somali [AOR = 2.8, 95% CI; 0.9–8.4]; Benishangul Gumuz [AOR = 0.82, 95% CI; 0.05–0.12]; SNNP [AOR = 4.3, 95% CI;1.7–11.2]), and type of NTD service provided by the facility (Schistosomiasis [AOR = 3.1, 95% CI; 1.4–6.7]). A statistically significant association was observed between the likelihood of facility readiness in terms of guidelines and the likelihood of the facility having a fixed cost for all types of services [AOR = 1.0, 95% CI; 0.0]. Regarding the medicine domain, the likelihood of readiness for HIV/AIDS facilities was associated with the facility type (hospitals [AOR = 1.0, 95% CI; 0.0]) and region (Amhara [AOR = 4.2, 95% CI; 1.5–11.3]); Dire Dawa [AOR = 0.4, 95% CI; 0.0.2–1.1]).

In malaria facilities, a statistically significant association was observed between the readiness in terms of trained staff and predictor variables such as region (Benishangul Gumuz [AOR = 1.0, 95% CI; 0.0], SNNP [AOR = 4.3, 95% CI; 1.7–11]), and type of NTD service provided by the facility (Schistosomiasis [AOR = 2.9, 95% CI; 1.4-6]). In terms of the guideline domain, the likelihood of readiness for a malaria facility was associated with the facility having a fixed cost for all types of services [AOR = 55.3, 95% CI; 2-157.3]). In terms of the medicine domain, the likelihood of facility readiness was associated with facility type (hospitals [AOR = 6.8, 95% CI; 2.6–17.7]) and the region (Amhara [AOR = 4.2, 95% CI; 1.6–11.1], Somali [AOR = 3.7, 95% CI; 1.1–12.1]), Harari [AOR = 2.0, 95% CI; 0.6–6.7], Dire Dawa [AOR = 0.5, 95% CI; 0.2–1.2]).

## Discussion

The recent study examined the percent service availability and level of readiness of TB, HIV/AIDS, and malaria health facilities to provide NTD care and its associated factors in Ethiopia. Hence, the findings of this study revealed a disparity in the proportion of NTD service availability, with relatively high availability of trained staff but an unsatisfactory scores in terms of guideline and essential medicine availability across the aforementioned health facilities in Ethiopia. Interestingly, none of those health facilities showed a high level of readiness to provide NTD services. Additionally, this study revealed significant associations between facility-specific readiness domains and independent variables such as facility type, region, presence of routine management meetings, types of NTD services, and fixed costs for services.

The relatively high percentage of facilities with at least one trained staff member for NTDs in Ethiopia mirrors findings from some other low- and middle-income countries. For instance, a study in Nigeria reported similar rates of trained staff availability across healthcare facilities for NTD management, [29]. However, disparities may exist in the availability of trained staff for specific NTDs, as observed in Ethiopia. This shows the importance of targeted training programs to address gaps in expertise for managing various NTDs. Similarly, findings from Uganda indicate challenges in staff training and healthcare capacity, affecting the availability of trained personnel for NTD management, [30]. A study in Sri Lanka revealed contrasting findings, with potential variations in healthcare infrastructure, workforce capacity, and government prioritization of NTD control programs, [31]. The low percentage of facilities equipped with guidelines for NTD management in Ethiopia reflects a common challenge in LMICs. Studies from countries such as Uganda [32], and Bangladesh, [33] have also reported deficiencies in standardized protocols for NTD treatment within healthcare facilities. This indicates the need for comprehensive policy frameworks and guidelines to streamline NTD management practices and ensure consistency in care delivery. In the present study, none of the facilities met the criteria for a high level of readiness, indicating significant gaps in access to NTD care services in Ethiopia. Given the existing epidemiological risks and high burden of TB, HIV/AIDS, malaria, and NTDs in the Ethiopian context, there is an urgent need to address the availability of important components among TB, HIV/AIDS, and malaria health facilities across the country regardless of facility type and managing authority, [9–11]. Otherwise, this may lead to an increased risk of coinfection with and treatment failure of other infectious diseases, hence fuelling the epidemic burden of infectious diseases in Ethiopia, [2–4].

The findings of this study revealed several factors associated with facility readiness for NTD treatment, including facility type, region, type of NTD service provided, and the presence of fixed costs for services in Ethiopia. Similar factors have been identified in studies from other countries. For example, a study in Nigeria found that facility type and geographical location significantly influenced the availability of NTD services, [29]. In contrast, a study in Uganda highlighted the importance of community health worker programs in enhancing access to NTD treatment in rural areas, [32]. These comparisons underscore the multifaceted nature of factors influencing facility readiness for NTD treatment, emphasizing the need for context-specific interventions. A study performed in Cambodia highlighted the influence of facility type on readiness, emphasizing the importance of adequately resourced healthcare facilities in delivering effective NTD interventions, [35]. A study in Burkina Faso demonstrated that facilities offering comprehensive NTD services tend to exhibit higher levels of readiness and capacity to manage these diseases effectively, [36]. Regional disparities in facility readiness for NTD treatment are evident in the Ethiopian context, with certain regions showing higher levels of readiness than others. Similar regional disparities have been observed in other countries. For instance, a study in Brazil illustrated significant variations in the availability of NTD services across different states, influenced by factors such as healthcare infrastructure and funding allocation, [34]. In Bangladesh, regional disparities in NTD prevalence and healthcare infrastructure have posed challenges to the delivery of integrated NTD services, [33]. These comparisons underscore the importance of targeted interventions to address regional disparities and improve access to NTD treatment in underserved areas. Studies in Nigeria, have shown disparities in facility readiness for NTD treatment between urban and rural areas as well as among different states. Challenges such as inadequate healthcare infrastructure, limited access to essential drugs, and disparities in healthcare financing contribute to these regional differences, [37].

The findings from this study underscore the need to address the gaps identified in the readiness of health facilities to offer treatment for NTDs in Ethiopia. Therefore, strengthening training and capacity-building programs for healthcare workers, enhancing access to essential medicines and diagnostics, promoting the integration of NTD services into existing healthcare delivery platforms, addressing regional disparities through targeted interventions, advocating for policy reforms to prioritize NTD control efforts, establishing robust monitoring and evaluation mechanisms, engaging local communities in NTD prevention and control efforts, and fostering research and innovation initiatives are important. These efforts aim to strengthen the healthcare system’s capacity to effectively diagnose, treat, and prevent NTDs, ultimately improving health outcomes for affected populations. For example, a study in Kenya highlighted the importance of strengthening primary healthcare systems and promoting community engagement to improve access to NTD services, [38]. These comparisons highlight the importance of context-specific approaches and multisectoral collaboration in addressing the complex challenges of NTD control and elimination.

This study has some limitations, but they are not sufficient to invalidate our study outcomes. Therefore, the findings of this study should be interpreted with caution due to the following limitations. First, as this study utilized a cross-sectional survey, the findings could not detect the long-term trends in the preparedness of TB, HIV/AIDS, and malaria facilities to manage NTDs. Hence, the drawbacks of the study design used in this analysis should be considered when interpreting our findings. Second, applying arbitrary cutoff thresholds might incorrectly classify the capability of TB, HIV/AIDS, and malaria facilities to provide NTD services.

This study has several strengths. First, to our knowledge, this is the first study to provide additional information regarding the extent to which Ethiopian TB, HIV/AIDS, and malaria facilities are available and prepared to provide NTD services. Second, the sample size was nationally representative, implying that the current results appropriately reflect the present status of health facility preparedness to integrate TB, HIV/AIDS, malaria, and NTD services in Ethiopia. Furthermore, considering the complicated sampling strategies utilized, the findings of this study were adjusted for the clustering effect and weighted to compensate for nonresponse and disproportionate sampling.

## Conclusions

In this study, TB, HIV/AIDS, and malaria health facilities had an overall unsatisfactory service availability and a lack of readiness to provide NTD care in Ethiopia. Additionally, factors such as facility type, region, presence of routine management meetings, types of NTD services provided, and fixed costs for services were significantly associated with facility readiness (domain specific). Given the high risk of coinfection and treatment failure of TB, HIV/AIDS, and malaria due to NTDs and their high burden in Ethiopia, the findings of this study further highlight the urgent need to consider developing and implementing a collaborative infectious disease care plan to integrate NTD services into the aforementioned facilities. Therefore, strengthening guideline dissemination, enhancing staff training, and improving medicine availability to enhance NTD service integration within existing healthcare frameworks is also critical in Ethiopia.

## Data Availability

We obtained administrative permission to access and use the dataset from ICF International, Rockville, Maryland, USA, through DHS program. Sequence data that supports the findings of this study is available on the repository: https://dhsprogram.com/data/available-datasets.cfm.
